# *trans*-Bis(acetonitrile-κ*N*)tetra­aqua­cobalt(II) tetra­chloridocobaltate(II)

**DOI:** 10.1107/S1600536809000750

**Published:** 2009-01-10

**Authors:** Violetta Patroniak, Maciej Kubicki

**Affiliations:** a Department of Chemistry, Adam Mickiewicz University, Grunwaldzka 6, 60-780 Poznań, Poland

## Abstract

In the title complex, [Co(CH_3_CN)_2_(H_2_O)_4_][CoCl_4_], the Co^II^ ions are octa­hedrally coordinated in the cation, with *trans*-disposed acetonitrile ligands, and tetra­hedrally coordinated in the anion. An extensive network of O—H(water)⋯Cl hydrogen bonds between cations and anions connects the ions into a three-dimensional network. The Co—Cl distances correlate with the number of hydrogen bonds accepted by the Cl atoms.

## Related literature

For background to our studies on new helical metal complexes, see: Stefankiewicz *et al.* (2008 ▶). There are only few examples of other bis­(acetonitrile)tetra­aqua complexes, these are mainly cobalt complexes: bis­(4,7-phenantroline) diperchlorate (Beauchamp & Loeb, 2002 ▶), dinitrate (Kopylovich *et al.*, 2001 ▶; Barnett *et al.*, 2002 ▶), dichloride monohydrate (Malkov *et al.*, 2003 ▶) and dibromide (Depree *et al.*, 2000 ▶), and one nickel complex, dibromide, has been reported (Assoumatine & Stoeckli-Evans, 2001 ▶). All these compounds have 1:2 composition. For a description of the Cambridge Structural Database, see: Allen (2002 ▶).
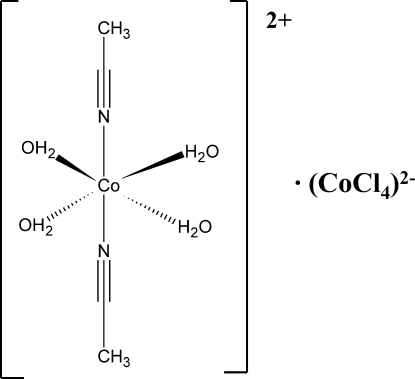


## Experimental

### 

#### Crystal data

[Co(C_2_H_3_N)_2_(H_2_O)_4_][CoCl_4_]
*M* *_r_* = 413.83
Orthorhombic, 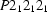

*a* = 7.0569 (4) Å
*b* = 12.3209 (8) Å
*c* = 17.9698 (12) Å
*V* = 1562.43 (17) Å^3^
*Z* = 4
Mo *K*α radiation
μ = 2.81 mm^−1^
*T* = 170 (2) K
0.2 × 0.2 × 0.2 mm

#### Data collection

Kuma KM-4-CCD diffractometer
Absorption correction: multi-scan (*CrysAlis RED*; Oxford Diffraction, 2007 ▶) *T*_min_ = 0.52, *T*_max_ = 0.57
6778 measured reflections
2619 independent reflections
2386 reflections with *I* > 2σ(*I*)
*R*_int_ = 0.027

#### Refinement

*R*[*F* ^2^ > 2σ(*F* ^2^)] = 0.064
*wR*(*F* ^2^) = 0.164
*S* = 1.03
2619 reflections
145 parameters
H-atom parameters constrained
Δρ_max_ = 3.41 e Å^−3^
Δρ_min_ = −0.58 e Å^−3^
Absolute structure: Flack (1983 ▶), 1006 Friedel pairs
Flack parameter: 0.28 (4)

### 

Data collection: *CrysAlis CCD* (Oxford Diffraction, 2007 ▶); cell refinement: *CrysAlis RED* (Oxford Diffraction, 2007 ▶); data reduction: *CrysAlis RED*; program(s) used to solve structure: *SHELXS97* (Sheldrick, 2008 ▶); program(s) used to refine structure: *SHELXL97* (Sheldrick, 2008 ▶); molecular graphics: *Stereochemical Workstation Operation Manual* (Siemens, 1989 ▶) and *Mercury* (Macrae *et al.*, 2008 ▶); software used to prepare material for publication: *SHELXL97*.

## Supplementary Material

Crystal structure: contains datablocks I, global. DOI: 10.1107/S1600536809000750/kp2200sup1.cif

Structure factors: contains datablocks I. DOI: 10.1107/S1600536809000750/kp2200Isup2.hkl

Additional supplementary materials:  crystallographic information; 3D view; checkCIF report

## Figures and Tables

**Table 1:** Selected bond lengths (Å)

Co1—O1*W*	2.085 (5)
Co1—O2*W*	2.076 (5)
Co1—O3*W*	2.067 (5)
Co1—O4*W*	2.088 (5)
Co1—N11	2.093 (6)
Co1—N21	2.106 (6)
Co2—Cl1	2.260 (2)
Co2—Cl2	2.2760 (19)
Co2—Cl3	2.2787 (19)
Co2—Cl4	2.3185 (18)

**Table 2:** Hydrogen-bond geometry (Å, °)

*D*—H⋯*A*	*D*—H	H⋯*A*	*D*⋯*A*	*D*—H⋯*A*
O1*W*—H1*WA*⋯Cl2^i^	0.90	2.34	3.185 (6)	155
O1*W*—H1*WB*⋯Cl3	0.90	2.40	3.250 (6)	157
O2*W*—H2*WA*⋯Cl4^ii^	0.90	2.29	3.153 (5)	159
O2*W*—H2*WA*⋯Cl4^ii^	0.90	2.29	3.153 (5)	159
O3*W*—H3*WA*⋯Cl3^i^	0.90	2.34	3.163 (6)	152
O3*W*—H3*WB*⋯Cl1^iii^	0.90	2.30	3.191 (6)	169
O4*W*—H4*WA*⋯Cl4^iv^	0.90	2.35	3.201 (5)	158
O4*W*—H4*WB*⋯Cl2^v^	0.90	2.36	3.199 (6)	155

Symmetry codes: (i) 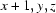; (ii) 
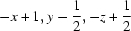
; (iii) 
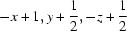
; (iv) 
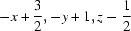
; (v) 
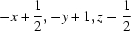
.

**Fig. 1.**
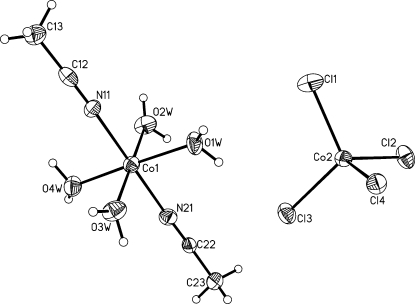
Anisotropic ellipsoid representation of compound I with atom labelling scheme (Siemens, 1989). The ellipsoids are drawn at the 50% probability level, hydrogen atoms are depicted as spheres of arbitrary radii. Hydrogen bonds are drawn as dashed lines.

**Fig. 2.**
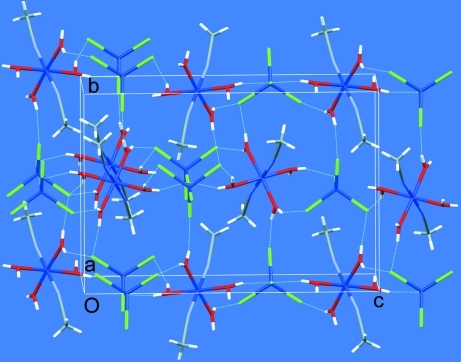
The crystal packing as seen approximately along a direction (Macrae et al., 2008). Hydrogen bonds are depicted as dashed lines.

## supplementary crystallographic information

### Comment

In the course of our studies on new helical metal complexes (*e.g.* Stefankiewicz *et al.*, 2008), we have prepared by chance the new *trans*-bis(acetonitrile-*N*)tetraaquacobalt(II) salt, with CoCl_4_ as the dianion. Surprisingly, there are only few examples of other salts of this dication, and in all six structures deposited in the CSDC (Ver. 5.29, Nov. 2007, Allen, 2002) the stoichiometry is 1:2, *i.e.* there are only monoanions.

In the cation the cobalt(ii) is octahedrally coordinated, with almost ideal geometry: the Co—N(O) distances are in the range 2.067 (5)–2.106 (5) Å, and the angles within the octahedron do not deviate more than 3° from the ideal values of 90° and 180°. The anion, as usual for CoCl_4_, forms a tetrahedron which geometry also deviates only slightly from the ideal values (see Fig. 1).

Both cations and anions are connected by the three dimensional network of O—H(water)···Cl hydrogen bonds. Interestingly, there is a correlation between the number of hydrogen bonds accepted by the Cl atom and the lengths of the Co—Cl bond: shorter the bond, less hydrogen bonds it accepts. The hydrogen bond network is built predominantly from the different rings, with 4 donors (4 different hydrogen atoms) and 2, 3, or 4 different chlorine atoms. The most important motifs found - not taking into account simple non-cyclic dimers of the form O—H···Cl - can be described with graph set symbols as *R*^4^_2_(10), *R*^4^_3_(10) and *R*^4^_4_(14).

### Experimental

The complex was prepared as described previously (Stefankiewicz *et al.*, 2008). The main product of this reaction was complex [Co(C_22_H_18_N_4_)(H_2_O)Cl](BF_4_).

### Refinement

Hydrogen atoms were located geometrically, in case of the water molecules on the basis of potential hydrogen bonds, and refined as the 'riding model' with U_iso_'s set at 1.3 times U_eq_'s of appropriate oxygen atoms. The relatively large values of residual electron density is probably an effect of unresolved twinning; the efforts on describing this twinning did not improve the model.

The Flack parameter (0.28 (4)) could suggest the possibility of a wrong absolute structure; however, the refinement of the inverted structure led to the higher values of the *R*-parameters (*R*(F) of 6.66% for observed and 7.13% for all reflections, wR2 is 16.35%) as well as for Flack parameter, which is 0.65 (4) in this case.

### Crystal data

**Table:**

[Co(C_2_H_3_N)_2_(H_2_O)_4_][CoCl_4_]	*F*(000) = 824
*M**_r_* = 413.83	*D*_x_ = 1.759 Mg m^−^^3^
Orthorhombic, *P*2_1_2_1_2_1_	Mo *K*α radiation, λ = 0.71073 Å
Hall symbol: P 2ac 2ab	Cell parameters from 5736 reflections
*a* = 7.0569 (4) Å	θ = 4–22°
*b* = 12.3209 (8) Å	µ = 2.81 mm^−^^1^
*c* = 17.9698 (12) Å	*T* = 170 K
*V* = 1562.43 (17) Å^3^	Prism, blue
*Z* = 4	0.2 × 0.2 × 0.2 mm

### Data collection

**Table:**

Kuma KM-4-CCD diffractometer	2619 independent reflections
Radiation source: fine-focus sealed tube	2386 reflections with *I* > 2σ(*I*)
graphite	*R*_int_ = 0.027
ω scans	θ_max_ = 25.0°, θ_min_ = 2.0°
Absorption correction: multi-scan (*CrysAlis RED*; Oxford Diffraction, 2007)	*h* = −8→8
*T*_min_ = 0.52, *T*_max_ = 0.57	*k* = −14→14
6778 measured reflections	*l* = −19→21

### Refinement

**Table:**

Refinement on *F*^2^	Secondary atom site location: difference Fourier map
Least-squares matrix: full	Hydrogen site location: inferred from neighbouring sites
*R*[*F*^2^ > 2σ(*F*^2^)] = 0.064	H-atom parameters constrained
*wR*(*F*^2^) = 0.164	*w* = 1/[σ^2^(*F*_o_^2^) + (0.131*P*)^2^ + 0.4597*P*] where *P* = (*F*_o_^2^ + 2*F*_c_^2^)/3
*S* = 1.03	(Δ/σ)_max_ < 0.001
2619 reflections	Δρ_max_ = 3.41 e Å^−^^3^
145 parameters	Δρ_min_ = −0.57 e Å^−^^3^
0 restraints	Absolute structure: Flack (1983), 1006 Friedel pairs
Primary atom site location: structure-invariant direct methods	Flack parameter: 0.28 (4)

### Special details

**Table:**

Geometry. All e.s.d.'s (except the e.s.d. in the dihedral angle between two l.s. planes) are estimated using the full covariance matrix. The cell e.s.d.'s are taken into account individually in the estimation of e.s.d.'s in distances, angles and torsion angles; correlations between e.s.d.'s in cell parameters are only used when they are defined by crystal symmetry. An approximate (isotropic) treatment of cell e.s.d.'s is used for estimating e.s.d.'s involving l.s. planes.
Refinement. Refinement of *F*^2^ against ALL reflections. The weighted *R*-factor *wR* and goodness of fit *S* are based on *F*^2^, conventional *R*-factors *R* are based on *F*, with *F* set to zero for negative *F*^2^. The threshold expression of *F*^2^ > σ(*F*^2^) is used only for calculating *R*-factors(gt) *etc*. and is not relevant to the choice of reflections for refinement. *R*-factors based on *F*^2^ are statistically about twice as large as those based on *F*, and *R*- factors based on ALL data will be even larger.

### Fractional atomic coordinates and isotropic or equivalent isotropic displacement parameters (Å^2^)

**Table:**

	*x*	*y*	*z*	*U*_iso_*/*U*_eq_	
Co1	0.75813 (15)	0.49124 (7)	0.11221 (5)	0.0234 (3)	
O1W	0.6109 (8)	0.4648 (5)	0.2112 (3)	0.0336 (13)	
H1WA	0.6469	0.4571	0.2590	0.044*	
H1WB	0.4977	0.4985	0.2113	0.044*	
O2W	0.6278 (8)	0.3582 (4)	0.0632 (3)	0.0369 (14)	
H2WA	0.6344	0.2855	0.0584	0.048*	
H2WB	0.5070	0.3775	0.0540	0.048*	
O3W	0.8873 (8)	0.6277 (4)	0.1558 (3)	0.0363 (14)	
H3WA	1.0017	0.6448	0.1747	0.047*	
H3WB	0.8287	0.6903	0.1440	0.047*	
O4W	0.8975 (7)	0.5205 (4)	0.0117 (3)	0.0336 (13)	
H4WA	0.9898	0.4702	0.0091	0.044*	
H4WB	0.8595	0.5339	−0.0353	0.044*	
N11	0.9845 (8)	0.3935 (5)	0.1465 (3)	0.0262 (13)	
C12	1.1077 (12)	0.3375 (6)	0.1583 (4)	0.0299 (18)	
C13	1.2670 (12)	0.2646 (6)	0.1711 (5)	0.0353 (18)	
H13A	1.3462	0.2612	0.1263	0.046*	
H13B	1.2192	0.1918	0.1828	0.046*	
H13C	1.3428	0.2916	0.2130	0.046*	
N21	0.5299 (9)	0.5881 (5)	0.0764 (4)	0.0274 (14)	
C22	0.4087 (10)	0.6463 (6)	0.0660 (4)	0.0229 (15)	
C23	0.2587 (12)	0.7229 (6)	0.0533 (5)	0.0389 (19)	
H23A	0.2599	0.7775	0.0930	0.051*	
H23B	0.1367	0.6848	0.0536	0.051*	
H23C	0.2767	0.7587	0.0051	0.051*	
Co2	0.16403 (13)	0.51515 (7)	0.35869 (5)	0.0222 (3)	
Cl1	0.3220 (3)	0.35571 (15)	0.36207 (13)	0.0393 (5)	
Cl2	−0.1531 (2)	0.48179 (16)	0.36180 (10)	0.0339 (5)	
Cl3	0.2520 (3)	0.61534 (15)	0.25785 (10)	0.0327 (5)	
Cl4	0.2382 (3)	0.61707 (13)	0.46316 (9)	0.0278 (4)	

### Atomic displacement parameters (Å^2^)

**Table:**

	*U*^11^	*U*^22^	*U*^33^	*U*^12^	*U*^13^	*U*^23^
Co1	0.0234 (5)	0.0248 (5)	0.0218 (5)	0.0022 (4)	−0.0002 (4)	−0.0008 (4)
O1W	0.030 (3)	0.050 (3)	0.021 (3)	0.003 (3)	0.008 (2)	0.006 (3)
O2W	0.034 (3)	0.031 (3)	0.046 (4)	0.001 (2)	−0.012 (3)	−0.004 (3)
O3W	0.036 (3)	0.029 (3)	0.043 (3)	0.002 (2)	−0.010 (3)	−0.008 (3)
O4W	0.028 (2)	0.044 (3)	0.029 (3)	0.005 (3)	0.000 (2)	0.007 (3)
N11	0.024 (3)	0.027 (3)	0.028 (3)	0.006 (3)	0.003 (3)	0.001 (3)
C12	0.034 (4)	0.031 (4)	0.025 (4)	−0.007 (4)	0.003 (3)	0.007 (3)
C13	0.032 (4)	0.039 (4)	0.035 (4)	0.006 (4)	−0.003 (4)	0.007 (3)
N21	0.029 (3)	0.022 (3)	0.031 (3)	−0.003 (3)	−0.001 (3)	−0.004 (3)
C22	0.019 (3)	0.023 (4)	0.026 (4)	−0.001 (3)	0.002 (3)	0.003 (3)
C23	0.028 (4)	0.034 (4)	0.055 (5)	0.008 (4)	0.011 (4)	0.011 (4)
Co2	0.0225 (5)	0.0211 (5)	0.0231 (5)	0.0001 (4)	−0.0011 (4)	0.0016 (4)
Cl1	0.0425 (11)	0.0256 (9)	0.0497 (12)	0.0065 (8)	−0.0087 (11)	−0.0011 (9)
Cl2	0.0243 (8)	0.0487 (11)	0.0288 (9)	−0.0083 (8)	−0.0021 (8)	0.0050 (9)
Cl3	0.0302 (9)	0.0409 (10)	0.0269 (9)	−0.0002 (10)	0.0028 (8)	0.0094 (8)
Cl4	0.0293 (9)	0.0270 (8)	0.0272 (9)	−0.0022 (8)	0.0009 (8)	−0.0032 (7)

### Geometric parameters (Å, °)

**Table:**

Co1—O1W	2.085 (5)	N11—C12	1.130 (10)
Co1—O2W	2.076 (5)	C12—C13	1.458 (11)
Co1—O3W	2.067 (5)	C13—H13A	0.9807
Co1—O4W	2.088 (5)	C13—H13B	0.9805
Co1—N11	2.093 (6)	C13—H13C	0.9806
Co1—N21	2.106 (6)	N21—C22	1.132 (9)
O1W—H1WA	0.9007	C22—C23	1.436 (10)
O1W—H1WB	0.9005	C23—H23A	0.9805
O2W—H2WA	0.9005	C23—H23B	0.9805
O2W—H2WB	0.9005	C23—H23C	0.9807
O3W—H3WA	0.9005	Co2—Cl1	2.260 (2)
O3W—H3WB	0.9009	Co2—Cl2	2.2760 (19)
O4W—H4WA	0.9007	Co2—Cl3	2.2787 (19)
O4W—H4WB	0.9008	Co2—Cl4	2.3185 (18)
			
O3W—Co1—O2W	177.1 (2)	Co1—O4W—H4WB	134.6
O3W—Co1—O1W	91.3 (2)	H4WA—O4W—H4WB	107.1
O2W—Co1—O1W	91.0 (2)	C12—N11—Co1	173.6 (6)
O3W—Co1—O4W	88.8 (2)	N11—C12—C13	178.2 (8)
O2W—Co1—O4W	88.7 (2)	C12—C13—H13A	109.6
O1W—Co1—O4W	178.1 (2)	C12—C13—H13B	109.3
O3W—Co1—N11	91.1 (2)	H13A—C13—H13B	109.5
O2W—Co1—N11	90.5 (2)	C12—C13—H13C	109.4
O1W—Co1—N11	92.2 (2)	H13A—C13—H13C	109.5
O4W—Co1—N11	89.7 (2)	H13B—C13—H13C	109.5
O3W—Co1—N21	89.6 (2)	C22—N21—Co1	171.1 (6)
O2W—Co1—N21	88.8 (2)	N21—C22—C23	178.2 (8)
O1W—Co1—N21	88.2 (2)	C22—C23—H23A	109.2
O4W—Co1—N21	89.9 (2)	C22—C23—H23B	109.3
N11—Co1—N21	179.2 (2)	H23A—C23—H23B	109.5
Co1—O1W—H1WA	133.6	C22—C23—H23C	109.8
Co1—O1W—H1WB	111.9	H23A—C23—H23C	109.5
H1WA—O1W—H1WB	107.2	H23B—C23—H23C	109.5
Co1—O2W—H2WA	143.4	Cl1—Co2—Cl2	109.11 (9)
Co1—O2W—H2WB	106.7	Cl1—Co2—Cl3	110.97 (9)
H2WA—O2W—H2WB	107.1	Cl2—Co2—Cl3	112.66 (8)
Co1—O3W—H3WA	136.8	Cl1—Co2—Cl4	109.74 (8)
Co1—O3W—H3WB	113.9	Cl2—Co2—Cl4	107.45 (8)
H3WA—O3W—H3WB	107.4	Cl3—Co2—Cl4	106.80 (7)
Co1—O4W—H4WA	105.4		

### Hydrogen-bond geometry (Å, °)

**Table:**

*D*—H···*A*	*D*—H	H···*A*	*D*···*A*	*D*—H···*A*
O1W—H1WA···Cl2^i^	0.90	2.34	3.185 (6)	155
O1W—H1WB···Cl3	0.90	2.40	3.250 (6)	157
O2W—H2WA···Cl4^ii^	0.90	2.29	3.153 (5)	159
O2W—H2WA···Cl4^ii^	0.90	2.29	3.153 (5)	159
O3W—H3WA···Cl3^i^	0.90	2.34	3.163 (6)	152
O3W—H3WB···Cl1^iii^	0.90	2.30	3.191 (6)	169
O4W—H4WA···Cl4^iv^	0.90	2.35	3.201 (5)	158
O4W—H4WB···Cl2^v^	0.90	2.36	3.199 (6)	155

Symmetry codes: (i) *x*+1, *y*, *z*; (ii) −*x*+1, *y*−1/2, −*z*+1/2; (iii) −*x*+1, *y*+1/2, −*z*+1/2; (iv) −*x*+3/2, −*y*+1, *z*−1/2; (v) −*x*+1/2, −*y*+1, *z*−1/2.

## References

[bb1] Allen, F. H. (2002). *Acta Cryst.* B**58**, 380–388. 10.1107/s0108768102003890 12037359

[bb2] Assoumatine, T. & Stoeckli-Evans, H. (2001). *Acta Cryst.* E**57**, m179–m180.

[bb3] Barnett, S. A., Blake, A. J., Champness, N. R. & Wilson, C. (2002). *Acta Cryst.* E**58**, m444–m446.

[bb4] Beauchamp, D. A. & Loeb, S. J. (2002). *Chem. Eur. J.* **8**, 5084–5088. 10.1002/1521-3765(20021115)8:22<5084::AID-CHEM5084>3.0.CO;2-8 12412059

[bb5] Depree, C. V., Ainscough, E. W., Brodie, A. M., Gainsford, G. J. & Lensink, C. (2000). *Acta Cryst.* C**56**, 17–18. 10.1107/s0108270199012159 10710651

[bb6] Flack, H. D. (1983). *Acta Cryst.* A**39**, 876–881.

[bb7] Kopylovich, M. N., Kukushkin, V. Yu., Guedes da Silva, M. F. C., Haukka, M., da Silva, J. J. R. F. & Pombeiro, A. J. L. (2001). *J. Chem. Soc. Perkin Trans. 1*, pp. 1569–1573.

[bb8] Macrae, C. F., Bruno, I. J., Chisholm, J. A., Edgington, P. R., McCabe, P., Pidcock, E., Rodriguez-Monge, L., Taylor, R., van de Streek, J. & Wood, P. A. (2008). *J. Appl. Cryst.* **41**, 466–470.

[bb9] Malkov, A. E., Fomina, I. G., Sidorov, A. A., Aleksandrov, G. G., Egorov, I. M., Latosh, N. I., Chupakhin, O. N., Rusinov, G. L., Rakitin, Yu. V., Novotortsev, V. M., Ikorskii, V. N., Eremenko, I. L. & Moiseev, I. I. (2003). *J. Mol. Struct.* **656**, 207–224.

[bb10] Oxford Diffraction (2007). *CrysAlis CCD* and *CrysAlis RED* Oxford Diffraction Ltd, Abingdon, England.

[bb11] Sheldrick, G. M. (2008). *Acta Cryst.* A**64**, 112–122. 10.1107/S0108767307043930 18156677

[bb12] Siemens (1989). *Stereochemical Workstation Operation Manual* Siemens Analytical X-ray Instruments Inc., Madison, Wisconsin, USA.

[bb13] Stefankiewicz, A. R., Wałęsa, M., Ciesielski, A., Patroniak, V., Kubicki, M., Hnatejko, Z., Harrowfield, J. M. & Lehn, J.-M. (2008). *Eur* *J* *Inorg* *Chem* pp. 2910–2920.

